# Quantifying and communicating radiation dose and risk from imaging procedures

**DOI:** 10.1002/jmrs.656

**Published:** 2023-01-20

**Authors:** Peter Thomas

**Affiliations:** ^1^ Medical Imaging Section Australian Radiation Protection and Nuclear Safety Agency Yallambie Victoria Australia

How much radiation will I receive from this imaging procedure? What is the associated risk? Such questions are often asked by patients and their guardians or carers. It is important for imaging facilities to be able to provide answers clearly and honestly and to be able to provide a context so that the benefits and risks of imaging can be understood and used to support informed decision‐making. Imaging facilities should assess the typical levels of radiation dose associated with commonly performed procedures. These data can be used to ascribe approximate estimates of risk. Information on dose and risk should be made available to practitioners and to patients and their guardians or carers to support discussions about the benefits and risks of imaging procedures.

A fundamental step is for a facility to be able to quantify the amount of radiation used for a particular imaging task. In Australia, this is a requirement of the Code for Radiation Protection in Medical Exposure (RPS C‐5).^1^ Key to this requirement is knowledge of the radiation output of equipment as a function of technique settings, or for nuclear medicine, knowledge of the administered activity, isotope, radiopharmaceutical and route of administration. Modern equipment typically includes devices to measure radiation output or software algorithms to calculate it, with the results displayed and archived in imaging data records. These should be supplemented by calibration and quality control measurements to ensure that reported output measures are accurate and consistent.^2^ Dose management software products are able to organise data records to provide statistical summaries of dose measures. Quantifying radiation doses given to patients requires a second step of applying the recorded output measures to models of the exposure geometry to determine absorbed doses to individual organs and tissues and compute effective dose. Methods vary from the use of summary conversion factors^3^ to software tools that model the actual exposure geometry more accurately, perhaps even extending to accounting for variation in the size of the patient.^4^ Effective dose is a useful summary metric, allowing general comparison of doses received when different parts of the body are exposed, although it does not account for some factors, such as age, sex and body size that are known to affect the risk of harm from radiation exposure.^5^, ^6^

In a research study published in this issue, Earl et al.^7^ have computed radiation doses for commonly performed general radiography procedures at a major paediatric hospital and similar work by the same group has been published previously for nuclear medicine procedures.^8^ These are excellent examples of a facility building a good understanding of patient exposures arising from their standard techniques. Other facilities are encouraged to do the same since practice may vary for a range of reasons such as equipment capabilities, patient cohort, disease presentation and the resources available for optimising technique factors. Wider availability of information on radiation doses across facilities and procedures would help to ensure that guidance material for patients and referring clinicians, such as the ‘Having a scan?’ information document on medical imaging exposures published by the Australian Radiation Protection and Nuclear Safety Agency^9^ for example, is appropriate and current. It can also foster and inform optimisation efforts as practitioners appreciate how doses at their site compare to those for equivalent procedures elsewhere.

In turning from dose to risk, we should acknowledge at the outset that there is considerable uncertainty, and some conjecture, regarding the true level of risk associated with the exposures encountered in diagnostic imaging. For most procedures, doses are well below the thresholds for tissue reactions and the resulting risks are probabilistic in nature, mainly risks of cancer, with a small component due to possible heritable effects. Current international guidance adopts the linear non‐threshold model in deriving estimates of risk at these low doses.^10^, ^11^ It is also important to note that such risk estimates do not apply to a specific person as many other factors may influence risks for a given individual. An important aspect is that risks of cancer arising from ionising radiation exposure have been found to vary with age at the time of exposure, with risks often (though not always) higher at younger ages and lower at older ages.^12^ Given the specific paediatric patient cohort, Earl et al.^7^ have applied the age‐specific risk factors compiled in the seventh Biological Effects of Ionizing Radiation report of the US National Research Council^13^ when estimating the lifetime cancer risks associated with the doses they have reported for their procedures.

Acknowledging the uncertainties, it is useful to consider such risk estimates in order‐of‐magnitude categories when evaluating benefit and risk, and for communication with patients and their carers. It is also important to contextualise the estimated risk through comparison with other risks and with the benefits that will arise from performing the procedure. Earl et al.^7^ provide an example of an information sheet developed to assist staff at their facility when discussing dose, risk and benefit with patients and their carers.

In an ideal future, all facilities would be recording and reviewing dose metrics for imaging procedures, summary data would be widely available for comparison, and patients and carers would have ready access to information about dose, risk and benefit that is easy to understand and helps support informed decision making.

## Conflict of Interest

The author declares no conflict of interest
